# Echocardiographic assessment of mitral regurgitation: discussion of practical and methodologic aspects of severity quantification to improve diagnostic conclusiveness

**DOI:** 10.1007/s00392-021-01841-y

**Published:** 2021-04-11

**Authors:** Andreas Hagendorff, Fabian Knebel, Andreas Helfen, Stephan Stöbe, Dariush Haghi, Tobias Ruf, Daniel Lavall, Jan Knierim, Ertunc Altiok, Roland Brandt, Nicolas Merke, Sebastian Ewen

**Affiliations:** 1 Department of Cardiology, Klinik und Poliklinik für Kardiologie, University of Leipzig, Liebigstraße 20, 04103 Leipzig, Germany; 2 Department of Cardiology, University of Berlin, Charité Universitätsmedizin Berlin, Campus Mitte, Medizinische Klinik mit Schwerpunkt Kardiologie und Angiologie, Charitéplatz 1, 10117 Berlin, Germany; 3 Department of Cardiology, Katholisches Klinikum Lünen Werne GmbH, St-Marien-Hospital Lünen, Altstadtstrasse 23, 44534 Lünen, Germany; 4 Kardiologische Praxisklinik Ludwigshafen, Akademische Lehrpraxis der Universität Mannheim, Ludwig-Guttmann-Strasse 11, 67071 Ludwigshafen, Germany; 5 Department of Cardiology, Center of Cardiology, Heart Valve Center, University Medical Center Mainz, University of Mainz, Langenbeckstrasse 1, 55131 Mainz, Germany; 6 Department of Cardiothoracic and Vascular Surgery, German Heart Center Berlin, Augustenburger Platz 1, Berlin, 13353 Germany; 7 Department of Cardiology, University of Aachen, Pauwelsstrasse 30, 52074 Aachen, Germany; 8 Department of Cardiology, Kerckhoff Heart Center, Benekestr. 2-8, 61231 Bad Nauheim, Germany; 9 Klinik für Innere Medizin III - Kardiologie, Angiologie und Internistische Intensivmedizin, Universitätsklinikum des Saarlandes, Kirrberger Str, IMED, 66421 Homburg, Germany

**Keywords:** Echocardiography, Mitral regurgitation, Quantification, Mitral regurgitant orifice area, PISA method, Regurgitant fraction

## Abstract

The echocardiographic assessment of mitral valve regurgitation (MR) by characterizing specific morphological features and grading its severity is still challenging. Analysis of MR etiology is necessary to clarify the underlying pathological mechanism of the valvular defect. Severity of mitral regurgitation is often quantified based on semi-quantitative parameters. However, incongruent findings and/or interpretations of regurgitation severity are frequently observed. This proposal seeks to offer practical support to overcome these obstacles by offering a standardized workflow, an easy means to identify non-severe mitral regurgitation, and by focusing on the quantitative approach with calculation of the individual regurgitant fraction. This work also indicates main methodological problems of semi-quantitative parameters when evaluating MR severity and offers appropriateness criteria for their use. It addresses the diagnostic importance of left-ventricular wall thickness, left-ventricular and left atrial volumes in relation to disease progression, and disease-related complaints to improve interpretation of echocardiographic findings. Finally, it highlights the conditions influencing the MR dynamics during echocardiographic examination. These considerations allow a reproducible, verifiable, and transparent in-depth echocardiographic evaluation of MR patients ensuring consistent haemodynamic plausibility of echocardiographic results.

**Graphic abstract:**

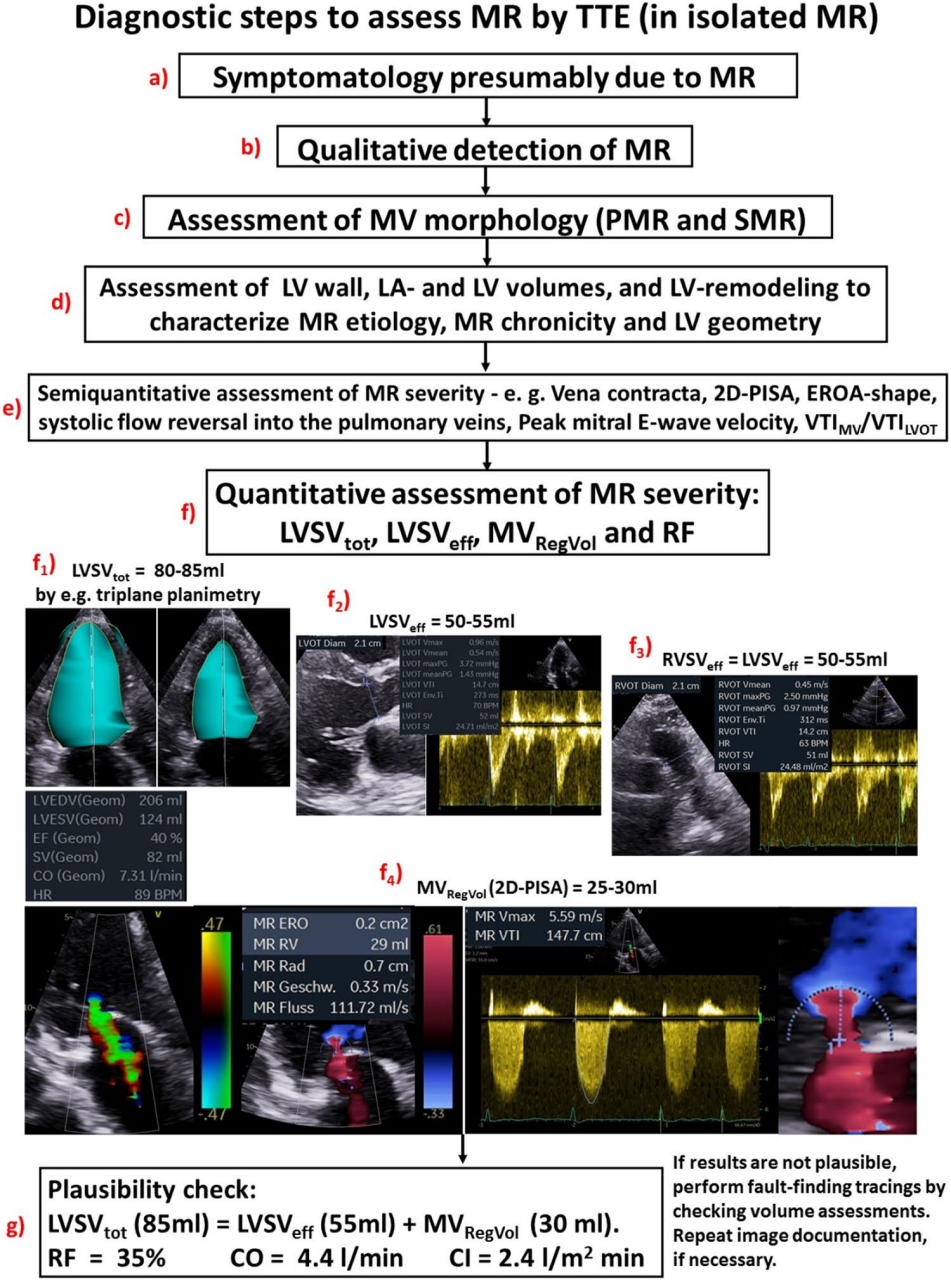

## Introduction

**Fig. 1:**
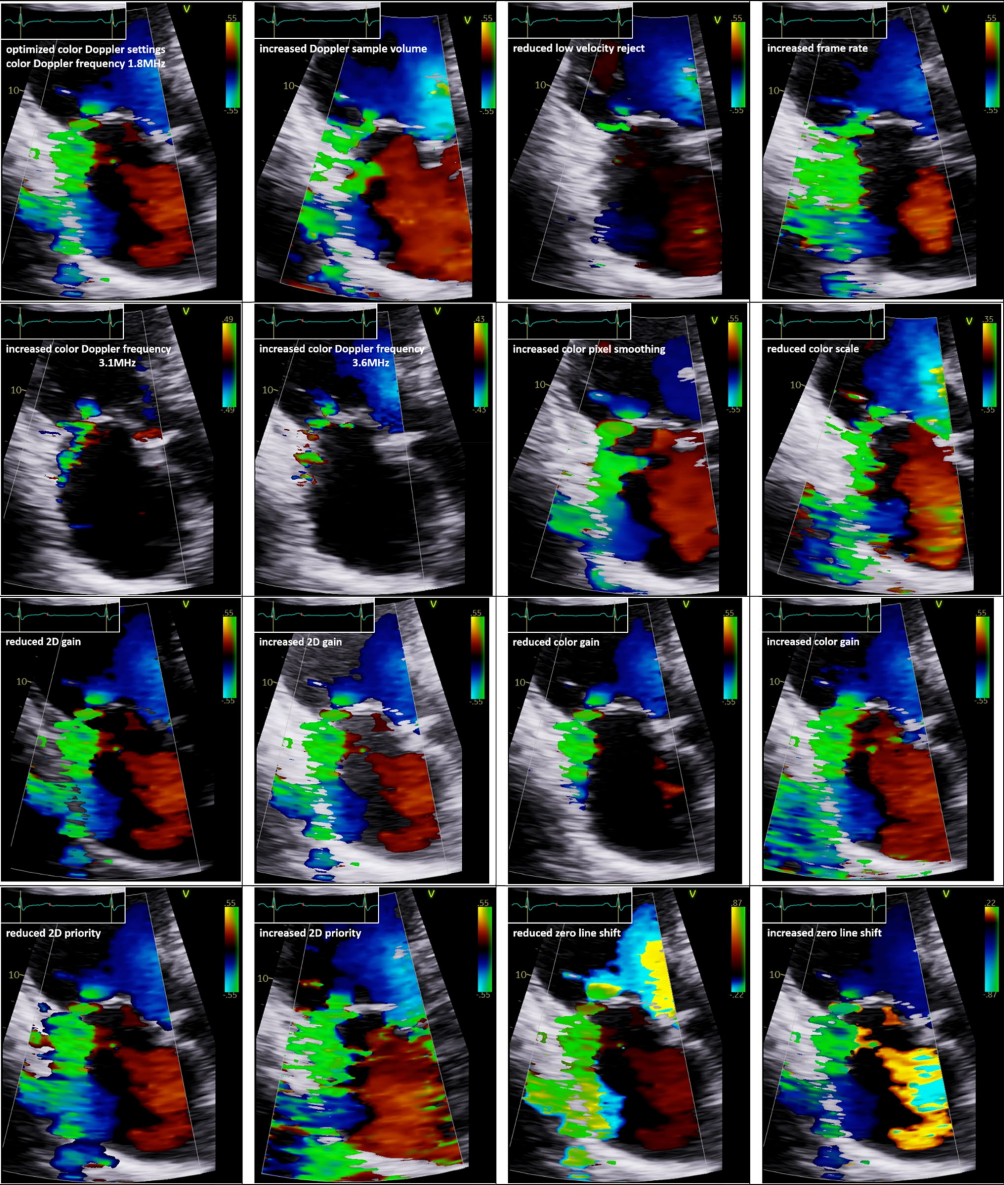
The methodological factors influencing color-coded flow phenomena (PISA, VC, jet area)—illustrated by optimal colour Doppler settings with 1.8 MHz Doppler frequency, increased Doppler sample volume, reduced low-velocity reject, increased frame rate, increased Doppler frequency with 3.1 and 3.6 MHz, increased colour pixel smoothing, reduced colour scale, reduced and increased 2D gain, reduced and increased colour gain, reduced and increased 2D priority, and reduced and increased zero line shift

The most frequently used tool for mitral regurgitation (MR) quantification in clinical practice is “eyeballing” of the colour flow jet area to differentiate between mild and severe MR [1]. This practice is primarily explained by its ease of use. However, it seems inadequate to solely use a qualitative diagnostic parameter to distinguish between mild, moderate, and severe MR [2–5]. As mentioned in recent recommendations, “eyeballing” of the MR jet area is misleading [3, 5, 6]. This is caused by its variations depending on ultrasound settings (Fig. 1), the different display of the jet area in respective sectional planes, and the haemodynamic variations influencing MR dynamics. In consequence, recent papers had eliminated this method in the respective tables [6, 7]. The key point statements—“The colour flow area of regurgitant jet is not recommended to quantify the severity of MR. The colour flow imaging should only be used for diagnosing MR. A more quantitative approach is required when more than a small central MR jet is observed” [3]—emphasize the necessity of a definite quantitative approach for grading MR severity.

At the same time, semi-quantitative and/or quantitative parameters, such as the 2D-PISA (proximal isovelocity surface area) method, are used by a minority of primary care physicians and cardiologists [1], whereas in clinical trials, it is the most frequently used method for MR quantification [8–11].

**Fig. 2:**
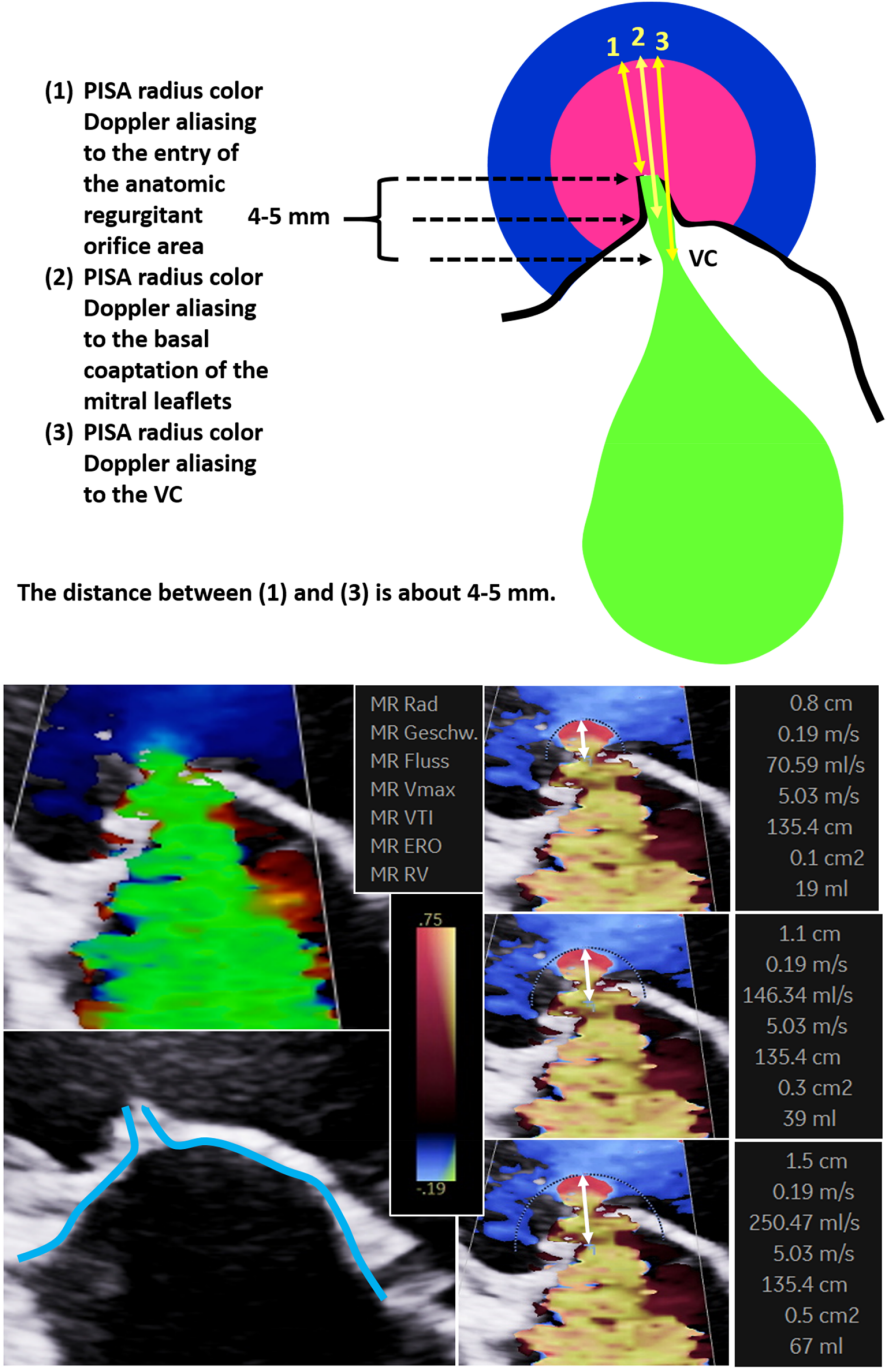
Limitations of the 2D-PISA method (PISA radius = *r*)—scheme of the proximal convergence areas and the proximal regurgitant flow phenomenon through the regurgitant orifice illustrating the importance of the accurate definition of the 2D-PISA radius. Example of regurgitant volume (MV_RegVol_) assessment using different 2D-PISA radii with equal velocity time integrals of regurgitant velocities (*r* = 8 mm, MV_RegVol_ = 19 ml; *r* = 11 mm, MV_RegVol_ = 39 ml; *r* = 15 mm, MV_RegVol_ = 67 ml)

Although recent recommendations describe the numerous limitations of the 2D-PISA method, making its use difficult, one key point message remains, namely “When feasible, the PISA method is highly recommended to quantify the severity of MR” [3]. However, the exact way of measuring of the 2D-PISA radius is unclear, as illustrated in Fig. 2. In the recent guidelines [5] “The radius of PISA is measured from the point of color Doppler aliasing to the VC (vena contracta)”. However, the 2D-PISA radius is illustrated in this recommendation [5], in the first description of the method [12], and in several other references [13] from the proximal convergence area to the ostium of the regurgitant orifice. This discrepancy is not clearly analysed in the literature [5, 13–15]—especially using modern colour Doppler technologies. Also, the impact of MR jet orientation is being debated controversially when using the 2D-PISA method. While some recommendations advise the use of the 2D-PISA in both central and eccentric jets [3, 16], others advise caution [15].

These two mainly used diagnostic features—the colour flow jet area and the 2D-PISA method—are complemented by additional semi-quantitative parameters, which eventuate in the “integrated approach” of MR quantification [3, 5, 6, 16]. However, all these semi-quantitative parameters have their limitations and can only be used in certain circumstances [13, 17–19]. A summary of the strengths and limitations of semi-quantitative parameters for grading of MR severity and the conditions when to apply or not to apply the respective parameters is particularized in Table 1. Considering the methodological challenges of the integrated approach, several concerns of semi-quantitative grading of MR severity should be considered to reduce the inter-observer variability to characterize more precisely and objectively MR severity. The dynamic nature of MR—especially with respect to loading conditions—cause a variability of MR quantification in clinical practice [20–22]. Thus, especially in secondary MR (SMR) recommendations favour the approach of MR assessment at compensated stage [16].

**Table 1:** Strengths, and limitations of the semi-quantitative parameters for grading MR severity focusing when to use or not to use the respective parameters

Semiquantitative parameter^a^	Strengths	Limitations	When to use or not to use
Valve morphology [3, 5, 23]	Easy to detect by TTE or TEE	Possibility of misinterpretation due to high heart rates	The only entity to imply severe MR is the rupture of a complete papillary muscle
LA and LV size [3, 5, 6]	LA and LV Enlargement are sensitive for chronic relevant MR Normal LV size excludes chronic relevant MR	Reliable results depend on standardization of sectional planes—thus, 3D volume assessment is preferred high inter-observer variability depending on image quality	Only if delineation of endocardial contours is practically possible If necessary, contrast echocardiography is recommended Quantitative assessment of LA and LV size is not reliable performed in foreshortening views and, if limited image quality is present
Vena contracta size [17, 18])	Easy to use, relatively independent of hemodynamic factors	Dependent on ultrasound settings, e.g., smoothing, low-velocity reject, 2D and color gain, etc. error-prone for eccentric jets	Mostly applicable in central jet formations using the parasternal long axis view Not reliable in the presence of eccentric jets especially in primary MR and in the region of the medial or lateral commissure if oblique sectional planes of the vena contracta (not perpendicular to the defect) are documented
2D-PISA-EROA; 2D-PISA-RegVol_MV_ size [3, 5, 6]	Possible to quantitatively assess EROA (lesion severity) and RegVol_MV_ with respects to methodologic accuracy	Underestimation of EROA and RegVol_MV_ by the elliptical shape of EROA Overestimation by improper labeling of the PISA radius PISA elongation by constrained flow field or eccentric jets, and by the dynamic nature of the MR Very limited if applied in eccentric jets—even using angle correction; limited by error-proneness of the PISA radius detection. Thus, high inter-observer variability	Only broadly applicable in central jet formation with flat PISAs (mostly to be observed in SMR Carpentier type 1 in patients with reduced LV function) Not applicable in eccentric PISAs (eccentric jet formation) and in elevated parabolic PISAs (constrained flow patterns) Not usable in multiple MR jets Non-applicable in late systolic dynamic MR (primary MR)
Shape of the EROA by 2D- and 3D-echocardiography size [24–26]	Applicable to detect individual changes of EROA using TEE Semilunar shape of EROA predictable for moderate or severe MR	Difficult to standardize the EROA in just one sectional plane due to its 3-dimensional shape—even using 3D techniques dependent on pixel size and ultrasound settings, not well validated in the literature	Only applicable in TEE; applicable to document individual changes of EROA in relation to hemodynamic factors Applicable to document acute treatment effects during intervention or surgery, diagnostic conclusiveness is limited by TTE color Doppler due to low spatial and temporal resolution Interpretation of EROA shape in TTE is very error-prone
Systolic flow reversal into the pulmonary veins size [27]	Simply to use and—if detectable—specific for severe MR	Dependent of flow direction of the regurgitant jet, on LA size and LA function, on LV contractility, and on hemodynamic factors as well as heart rhythm	Only well applicable, if sinus rhythm is present, if regurgitant jet enters the right pulmonary veins in TTE, and the left pulmonary veins in TEE, if LA size is normal or only mildly enlarged, and if LV contractility is normal or mildly reduced Thus, not applicable in severely enlarged LA, in severe LV dysfunction and during atrial fibrillation
Intensity of the regurgitant velocity signal using continuous wave (cw) Doppler and cw-jet profile size [3, 5, 6]	Easy to document and to interpret A triangular cw-jet profile indicates relevant MR severity	The regurgitant flow velocities should be recorded during the complete systole, that implies correct Doppler delineation during the complete heart cycle	The interpretation and jet profile can only be interpreted if acquisition of the cw-spectrum is methodically correct Thus, this method is only accepted as qualitative parameter due to its methodologic limitations [3, 5] Not applicable, if cw- alignment with blood flow is not verified, especially in eccentric regurgitant jets Proper Doppler alignment is almost always feasible by TTE
Peak mitral E-wave velocity, peak mitral A-wave velocity size [6]	Easy to document by transmitral pulsed wave (pw) Doppler Vmax of E wave < 1 m/s often indicates non-relevant MR Increased A-wave excludes relevant MR	The correct interpretation depends on correct position of pw-sample volume Atrial fibrillation	Applicable, if the orifice area of the mitral valve is normal, if mitral annulus diameter is normal (a.p.-diameter < 35 mm) Limited diagnostic value in atrial fibrillation and severe diastolic dysfunction Not applicable in mitral stenosis Not applicable in severe mitral annulus dilatation
The ratio of transmitral velocity time integral (VTI_MV_) and flow velocity time integral within the LV outflow tract (VTI_LVOT_) (VTI_MV_/VTI_LVOT_) size [3, 28]	Easy to determine using pw-Doppler spectra	Diagnostic value depends on the accuracy of the positions of the sample volumes, which should be located at the tip of the MV leaflets and in the LVOT considering optimal alignment of the ultrasound beam with blood flow	Only applicable, if mitral annulus is not severely dilated and normal mitral valve morphology, leak-tight aortic valve and/or and/or atrial fibrillation is present Thus, not applicable in severe mitral annulus dilatation, in mitral stenosis, and in aortic regurgitation Error-prone in atrial fibrillation

Morphological parameters like papillary muscle rupture, LA and LV size, as well as the semi-quantitative parameters vena contracta, 2D-PISA-EROA and 2D-PISA-MV_RegVol_, the shape of the EROA, systolic flow reversal into the pulmonary veins, intensity of the regurgitant velocity signal using continuous wave Doppler and the jet profile, peak mitral E- and A-wave velocity, and the ratio of transmitral velocity time integral (VTI_MV_) and flow velocity time integral within the LV outflow tract (VTI_LVOT_) (VTI_MV_/VTI_LVOT_) are introduced

^a^The color Doppler jet area is not listed in the table of semi-quantitative parameters, because this parameter is solely useful for the qualitative detection of a mitral regurgitation, but is not recommended for grading the MR severity (Lancellotti et al. 2010 and 2016). Specific types of jet morphology, e.g., Coanda phenomenon, explain the defect morphology and give cause for quantitative assessment of MR severity

The assessment of MR and the grading of its severity remain challenging today. It is the objective of this work to present tools for an in-depth analysis of the MR, taking practical, methodological, and pathophysiological aspects into consideration. To improve diagnostic conclusiveness the quantitative approach of MR assessment by determining left ventricular (LV) total and effective stroke volume (LVSV_tot_, LVSV_eff_), regurgitant volume at the mitral valve level (MV_RegVol_) and regurgitant fraction (RF) is highlighted.

## A proposal for a standardized workflow of the echocardiographic MR assessment

A standardized workflow during the echocardiographic examination and the patient`s visit is necessary to ensure a reproducible and verifiable MR assessment as well as documentation of treatment effects in MR patients. Patient`s characteristics and clinical parameters must be considered for therapeutic decision-making. Indexing of several echocardiographic parameters is based on body height, body weight, and surface area. Systemic blood pressure enables estimation of LV afterload. Clinical symptoms and their progression as well as alterations of echocardiographic parameters with disease progression are important to decide the necessity of therapeutic interventions. At last, age and comorbidities are not influencing MR severity, but are important to estimate the individual patients’ risk. Multiple factors cause differences in MR severity in the same patient at different time points, e.g., cardioversion of atrial fibrillation (AF) into sinus rhythm, resynchronisation therapy in patients with left bundle branch block (LBBB), optimized medical treatment (OMT) in heart failure, or revascularization in myocardial ischemia. To ensure comparability of echocardiographic investigations MR assessment should be performed according to recent recommendations at compensated stage [16].

**Fig. 3:**
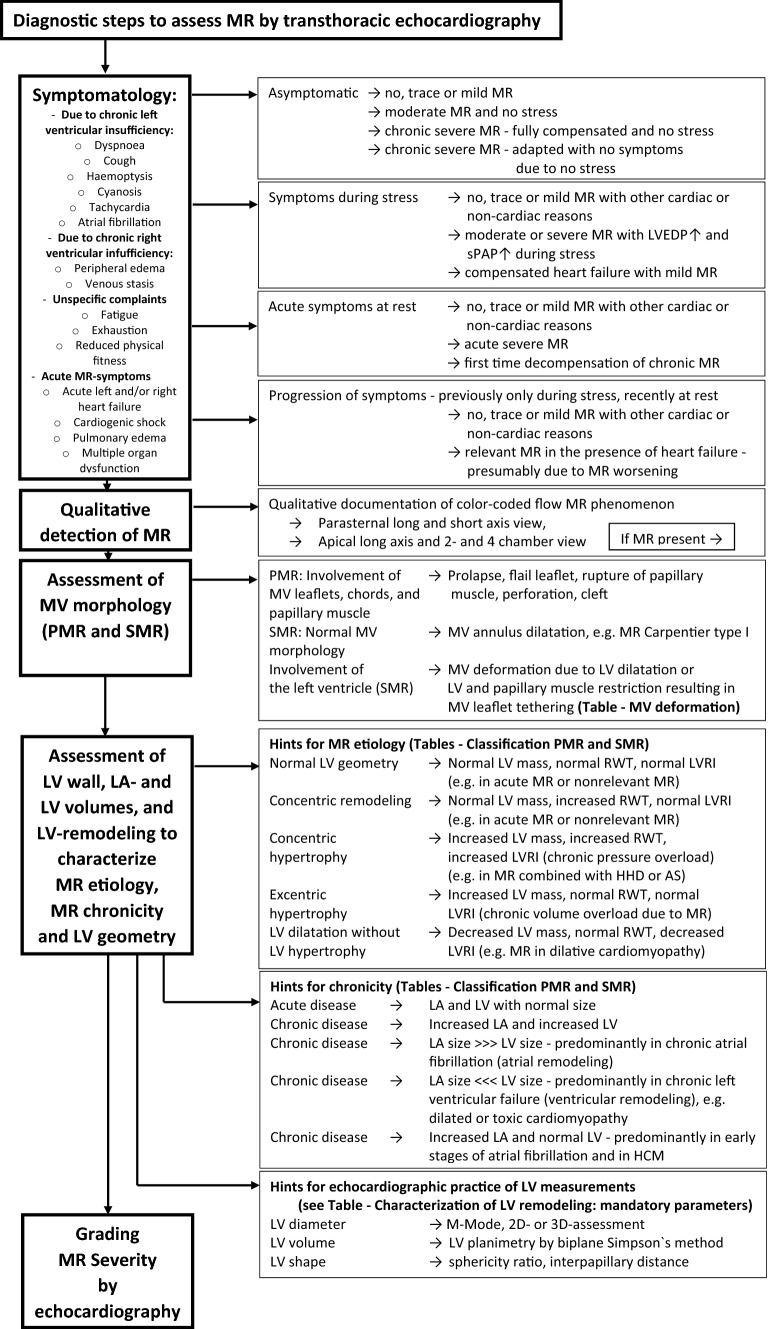
Scheme to illustrate the diagnostic steps to assess MR by TTE: The first step includes the interpretation of clinical symptoms and the chronicity of the underlying disease in the context of MR with different severity. The second step is the qualitative detection of MR. The third step is the analysis of MV morphology and the differentiation between PMR and SMR. The fourth step is the assessment of LV wall, LA- and LV volumes as well as LV size and LV remodelling to get insights into MR etiology, MR chronicity, and LV geometry. The last step is the grading of MR severity. *HOCM *hypertrophic cardiomyopathy, *LA *left atrial, *LV* left ventricular, *LVEDP LV* end-diastolic pressure, *LVRI LV* remodelling index, *MR *mitral regurgitation, *MV *mitral valve, *PMR *primary MR, *RWT *relative wall thickness, *SMR *secondary MR, *sPAP *systolic pulmonary arterial pressure

**Fig. 4:**
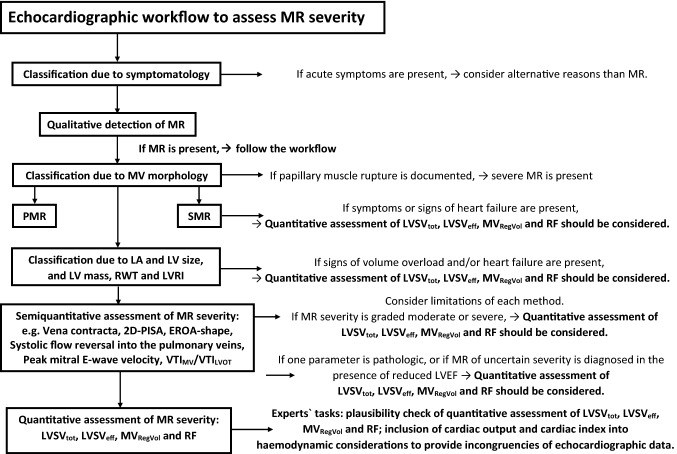
Scheme to illustrate the echocardiographic workflow to assess MR severity: After interpretation of symptomatology with respect to the causal relationship to the MR qualitative MR detection results in MR classification due to the MV morphology. Echocardiographic parameters of LA and LV size and LV wall thickness characterize loading conditions and enable to distinguish between pressure or volume overload and between compensated or decompensated conditions. The assessment of MR severity starts with the integrated approach and the analysis of semi-quantitative parameters. The final experts’ task of analysis of MR severity is the quantitative assessment of LVSV_tot_, LVSV_eff_, MV_RegVol_, and RF as a plausibility check. At every level of the assessment of MR severity expert consultation as well as the quantitative analysis of MR severity should be considered with respect to severe symptoms, signs of volume overload and heart failure as well as incongruent results by the grading of MR severity by the semi-quantitative approach. *2D *two-dimensional, *EROA *effective regurgitant orifice area, *LA *left atrial, *LV *left ventricular, *LVOT LV* outflow tract, *LVRI LV* remodelling index, *LVSV*_*eff*_ effective LV stroke volume, *LVSV*_*tot*_ total LV stroke volume, *MR* mitral regurgitation, *MV* mitral valve, *MV*_*RegVo*l_ regurgitant MV volume, *PISA* proximal isovelocity surface area, *PMR* primary MR, *RF* regurgitant fraction, *RWT* relative wall thickness, *SMR* secondary MR, *VTI* velocity time integral

**Fig. 5:**
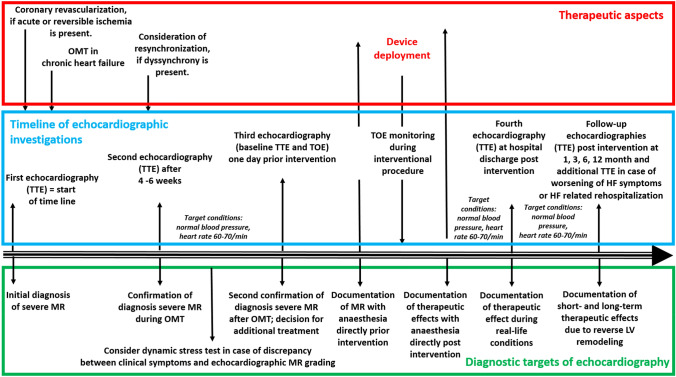
Proposal for standards of echocardiographic timing in MR patients. The scheme illustrates a potential timeline of echocardiographic investigations during MR treatment. The upper red box presents the therapeutic aspects and strategies, the mid blue box presents the proposed time points of echocardiographic investigations—especially focusing on secondary mitral regurgitation (SMR)-, the bottom green box illustrates the diagnostic targets of the respective echocardiographic investigations. *LV* left ventricular, *MR *mitral regurgitation, *OMT* optimized medical treatment, *TOE* transoesopageal echocardiography, *TTE* transthoracic echocardiography

The echocardiographic examination should consider and interpret the clinical symptoms, and the individual patient`s factors in relation to the presumed valvular defect (Figs. 3, 4). After qualitative MR detection by Doppler techniques, the next diagnostic steps by echocardiography should be the assessment of mitral valve (MV) morphology, LV wall thickness, left atrial (LA), and LV volume as well as LV shape and remodelling, prior to grading MR severity (Figs. 3, 4). Thereafter, a semi-quantitative MR assessment is advised, which should be followed by a quantification of MR severity, if moderate or severe MR is being suspected, or if severity of MR remains unclear (Fig. 4). Every changes of MR severity documented by repetitive standardized echocardiography should be noted to enable reliable conclusions about the respective treatment effects. Figure 5 depicts a recommended timeline for performing echocardiographic examinations in patients with significant MR who are considered for interventional/surgical treatment of MR.

## The rationale for the stepwise workflow to assess MR severity to implement the causal relationships between clinical complaints, disease progression, and echocardiographic characteristics into the “integrated approach”

Identifying a causal relationship between clinical symptoms and MR might facilitate the interpretation of echocardiographic results in MR patients. However, symptoms as well as echocardiographic findings depend on chronicity of the disease progress. Acute MR is normally linked to severe symptoms, smaller LA and LV cavities, and severe PH, whereas chronic MR is linked to mild symptoms, larger LA and LV cavities, and different secondary PH severity. Due to this pathophysiological complexity, all possible morphologic variations of LA and LV size can be observed in clinically relevant MR.

If MR is qualitatively detected by Doppler techniques—e.g., colour flow Doppler—MV morphology should help differentiating between primary MR (PMR) and secondary MR (SMR) [29]. This classification focusses on morphological defects of the MV apparatus (PMR) and on secondary MV alterations induced by underlying LV diseases. Thus, structural involvement of the MV apparatus characterizes PMR and LV dilatation and/or LV dysfunction SMR. Pathologies of the leaflets or alterations of the intricate anatomy of the MV apparatus are causes of PMR, failure of MV leaflet coaptation due to MV annulus dilatation, increased leaflet tethering, and/or papillary muscle (PM) restriction are causes of SMR [3–6, 30, 31]. Furthermore, Carpentier’s classification scheme according to leaflets mobility [32] considers functional aspects of the MV leaflets.

The pathophysiological understanding of cardiac alterations in MR requires a morphological characterization of the cardiac cavities [3–6, 16]. Both, PMR and SMR, impose a volume load on the left ventricle and the left atrium. LV dilatation increases MV tethering forces, while LV dysfunction reduces MV closing forces, both driving factors of SMR [33]. SMR resulting from predominant mitral annular dilatation is increasingly being recognized as SMR induced by atrial remodelling [34]. The volume load in chronic PMR and SMR further aggravates LV dilatation to accommodate for the MV_RegVol_ and to maintain LVSV_eff_. LV function is preserved in the compensated state in PMR, but declines in a decompensated condition. In the decompensated state, MV_RegVol_ itself is a pathophysiological driver that contributes to the disease progress with concomitant increase of LV end-diastolic pressure (LVEDP) and secondary pulmonary hypertension (PH) [35, 36]. LV ejection fraction (LVEF) overestimates LV function in MR. Forward LVEF = LVSV_eff_/LV end-diastolic volume (LVEDV) seems to represent more reliably LV function than global LVEF in MR [37, 38]. Hence, MR severity relative to LV remodeling has been proposed [16, 39, 40]. In consequence, LV function, LV remodelling, and global haemodynamics often differ between PMR and SMR. Thus, LV wall thickness, LV mass, LV mass index, LV diameter, LV volume, LVEF, as well as LA volume and LA volume should be measured by echocardiography to characterize LV geometry, e.g., concentric remodelling, and concentric and eccentric LV hypertrophy [41]. Relative wall thickness (RWT) and LV mass should be measured using the posterior wall. Considering clinical symptoms and chronicity of the underlying diseases in relation to the specific echocardiographic findings, an extended MR classification is proposed for PMR and SMR patients. Five subtypes can be differentiated in PMR (Table 2). Furthermore, seven subtypes can be differentiated in SMR (Table 3) with respect to symmetric LV remodelling, asynchrony of LV contraction, regional myocardial injury, asymmetric LV hypertrophy, LV stiffening, and LA remodelling [3, 5].

**Table 2:**
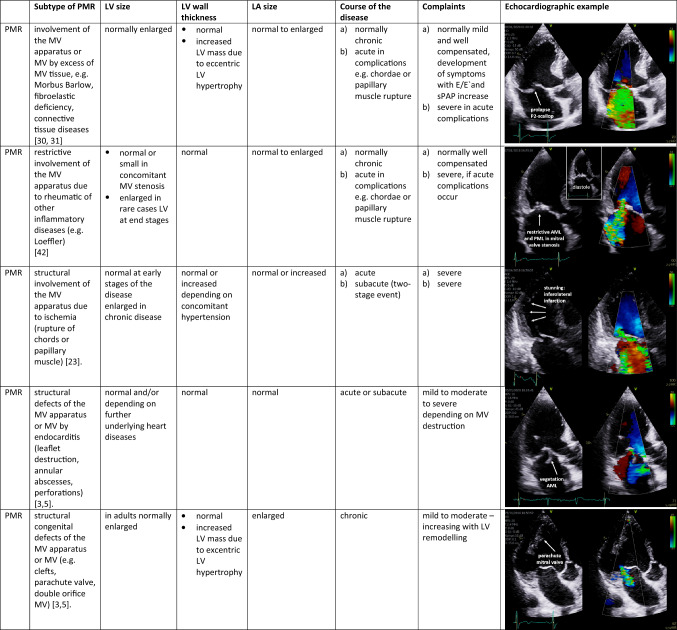
Proposal to classify primary mitral regurgitation (PMR) more in detail with respect to specific echocardiographic findings, the chronicity of the underlying diseases, and the clinical complaints of the patients

PMR subtypes are characterized by description of left-ventricular (LV) size, LV wall thickness, left atrial (LA) size, the course of the disease, and one respective echocardiographic example

*MV *mitral valve, *sPAP *systolic pulmonary arterial pressure

**Table 3:**
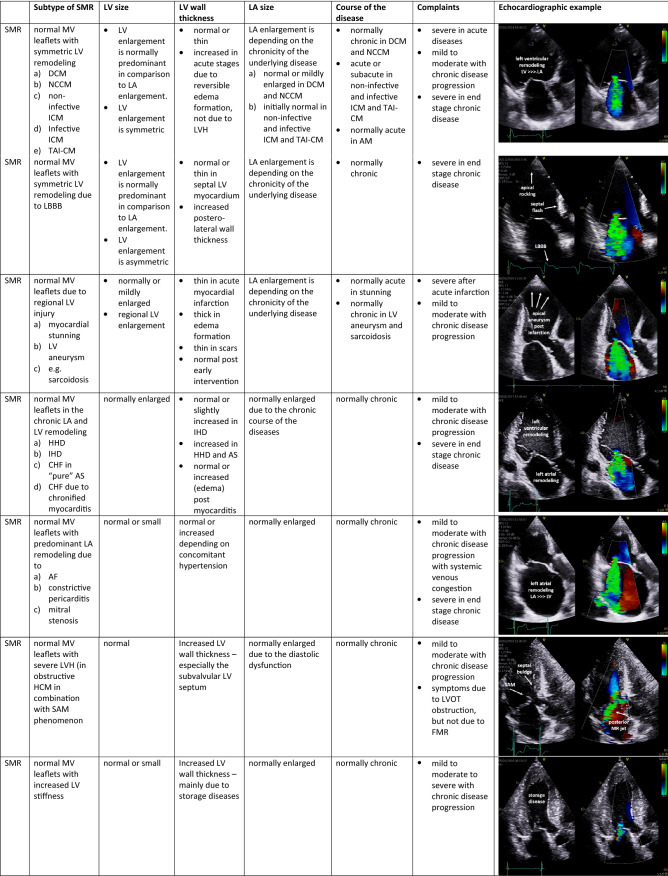
Proposal to classify secondary mitral regurgitation (SMR) more in detail with respect to specific echocardiographic findings, the chronicity of the underlying diseases, and the clinical complaints of the patients

PMR subtypes are characterized by description of left ventricular (LV) size, LV wall thickness, left atrial (LA) size, the course of the disease and one respective echocardiographic example

*AF *atrial fibrillation, *AM *acute myocarditis, *AS *aortic valve stenosis; *CHF* chronic heart failure, *DCM* dilated cardiomyopathy, *HCM *hypertrophic cardiomyopathy, *HHD *hypertensive heart disease, *ICM *inflammatory cardiomyopathy, *IHD *ischemic heart disease, *LBBB *left bundle branch block, *LVH *left-ventricular hypertrophy, *LVOT *left-ventricular outflow tract, *MV *mitral valve, *NCCM *non-compaction cardiomyopathy, *SAM *“systolic anterior movement”, *TAI-CM *tachyarrhythmia-induced cardiomyopathy

Thus, one target of paramount importance is to characterize cardiac remodelling due to MR effects, which implies the specific assessment of LV [43–45] and LA geometry by echocardiography [46]—especially in SMR patients [3, 5, 6]. Despite recent technical improvements in echocardiography and automated features to analyze LA and LV volumes and function, conventional 2D echocardiography remains the current standard and enables the assessment of relevant cardiac parameters as illustrated in Table 4. Linear internal 2D measurements of LV diameters and LV wall thickness as well as LV volume measurements by 2D planimetry are still used in clinical practice [5, 41, 47]—especially for calculation of LV mass [41]. 3D approaches for LV mass determination are preferably recommended [48]. The sphericity ratio and sphericity index, interpapillary muscle distance, the anterior–posterior and medial–lateral PM displacement, and the length between the PM bulges and the respective contralateral MV annulus should be considered for characterization of LV remodelling [3, 49–51]. Furthermore, LV remodelling with disease progression or reverse LV remodeling during treatment can be assessed by monitoring LV geometry [3, 51]. LA volume measurement by 2D planimetry of the maximum LA area in the 2- and 4-chamber view (2-ChV, 4-ChV) or using 3D echocardiography is preferred [48]. The progression of LA and LV volumes and reduction of LVEF during follow-up examinations are helpful to determine haemodynamically significant deterioration even in MR patients classified as clinically not severe.

**Table 4:**
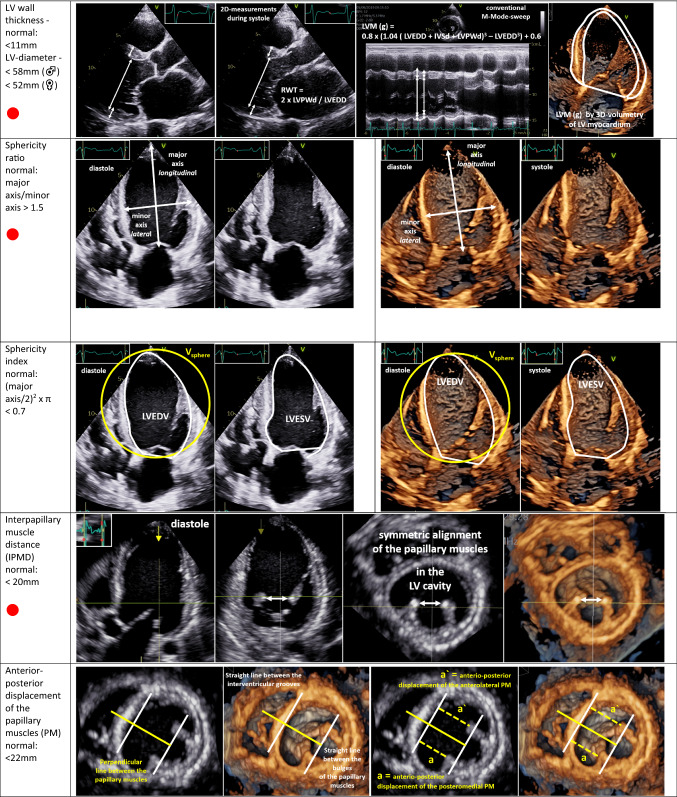

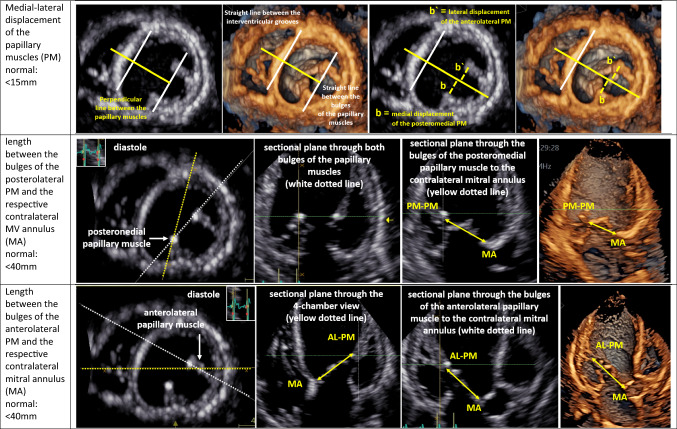
Echocardiographic parameters characterizing left-ventricular (LV) remodelling in MR patients using conventional 2D echocardiography or 3D TTE

In the first column, the echocardiographic target parameters are listed including the normal ranges: LV wall thickness and relative wall thickness (RWT), LV diameter, LV mass (LVM), sphericity ratio, sphericity index, interpapillary muscle distance (IPMD), anterior–posterior and medial–lateral displacement of the papillary muscles (PM), as well as the length between the bulges of the posterolateral or anterolateral PM and the respective contralateral MV annulus (MA). The parameters recommended as mandatory [3, 5] are marked with **●**. The respective images illustrate the assessment of the respective parameters in 2D sectional planes or within 3D data sets

The second target is the analysis of MV morphology by echocardiography. Due to the complexity of the MV apparatus, 3D image acquisition has become an indispensable tool of echocardiographic MV assessment [48, 52–55]. However, conventional 2D echocardiography enables the measurement of specific parameters characterizing pathologies of MV morphology. MV degeneration can be identified by the presence of intensified echo-densities due to thickening and calcification of the MV annulus [42]. MV prolapse is characterized by systolic displacement of a leaflet by ≥ 2 mm overriding the annular plane into the LA [3, 52]. Rupture of the primary chordae, or ultimately of a PM, causes flail of the leaflet into the LA and is usually associated with severe MR. Analysis of MV involvement in endocarditis should include size of vegetations, presence of abscesses, aneurysms, or perforations [56]. Congenital MV defects, e.g., clefts, can be uncovered in the 3D TOE, favouring definitively this modern technology [55]. MV deformation due to LV remodelling in SMR should be assessed by measurement of MV annulus, coaptation distance/gap, coaptation length/height, as well as coaptation depth and tenting height (Table 5) [3, 5, 57]. The tenting area (area between the MA and the leaflets during systole) of ≥ 2.1 cm^2^ is a pathologic finding due to tethering in SMR [3–5]. MV analysis in SMR should be completed by the assessment of the anterior/medial and posterior/posterolateral tethering angle (Table 5) [3, 5]. As pathophysiology of MR is a constant and complex interplay between initial pathology and further propagation of the disease by volume overload, coexistence between PMR and SMR can be observed and should be labeled as mixed origin.

**Table 5:**
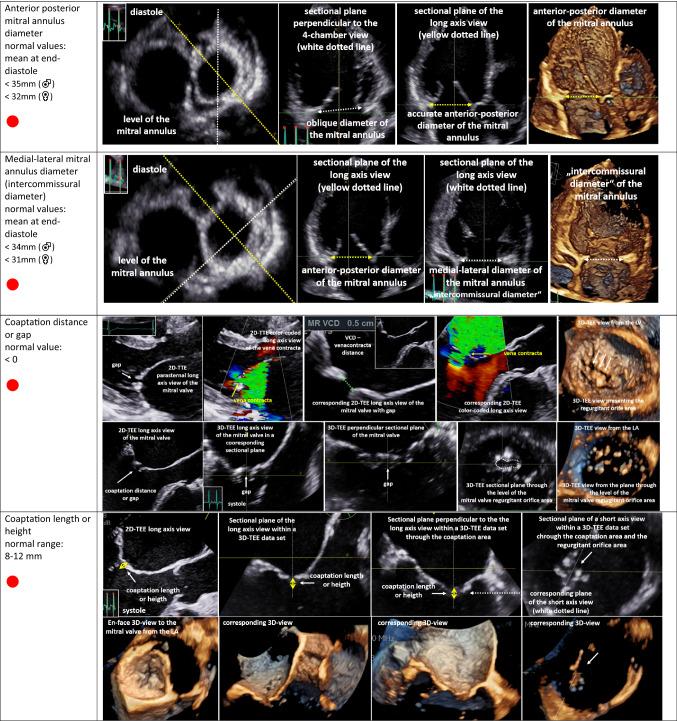

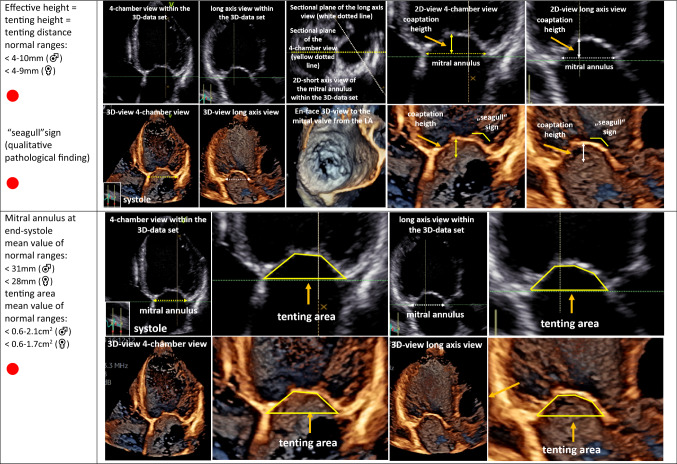

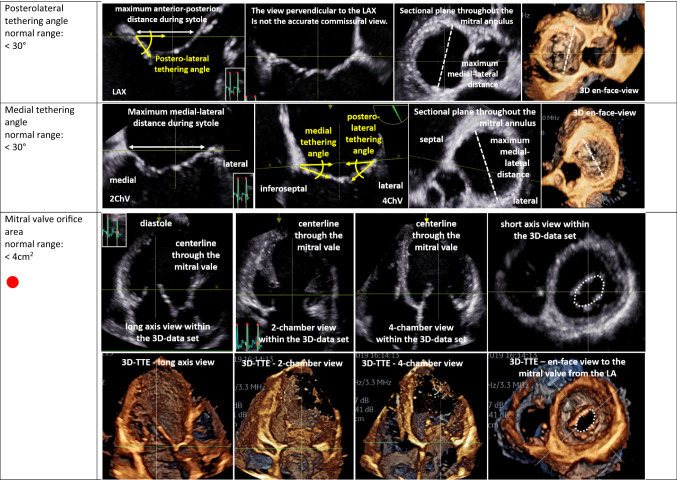
Echocardiographic parameters characterizing mitral valve (MV) deformation in SMR patients using conventional 2D echocardiography or 3D TTE

In the first column, the echocardiographic target parameters are listed including the normal ranges: anterior–posterior and medial–lateral MV annulus diameter (intercommissural diameter) at early diastole, coaptation distance or gap, coaptation length or height, effective height, tenting height or tenting distance, the “seagull” sign, MV annulus diameter at end-systole, posterolateral and medial tethering angle, and mitral valve orifice area. The parameters recommended as mandatory [3, 5] are marked with **●**. The respective images illustrate the assessment of the respective parameters in 2D sectional planes or within 3D data sets

## The rationale to implement a quantitative MR assessment to characterize MR severity

The echocardiographic workflow of grading MR severity (Figs. 3, 4) starts with a semi-quantitative MR assessment and serves two goals. First, all non-severe MR should be detected, preventing unnecessary and time-consuming further evaluation. For example, when sinus rhythm is present, an a-wave dominant inflow pattern into the LV using Doppler interrogation above the MV excludes severe MR. Also, a dominant inflow during systole from the pulmonary veins into the LA cannot be observed in severe MR. Finally, a normal LA volume is not found in chronic severe MR. These and other semi-quantitative parameters, along with their strengths, limitations, and appropriateness are listed in Table 1. In-depth quantitative evaluation should be initiated in cases if MR classification remains unclear.

**Fig. 6:**
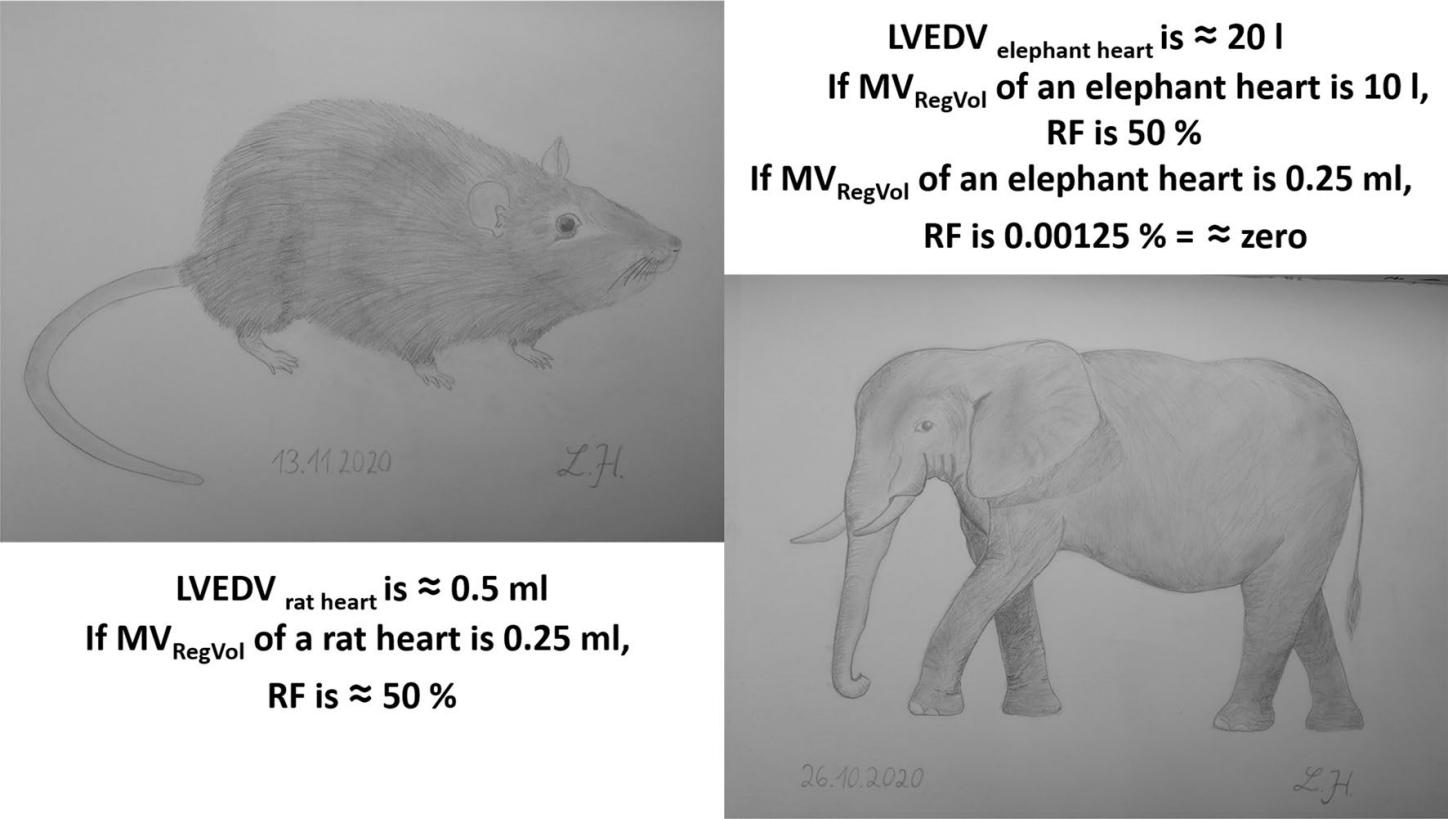
Illustration of the interspecies differences of regurgitant volume in relation to total stroke volume (LVSV_tot_). The normal LVSV_tot_ of a rat heart is about 0.5 ml [61] resulting in a regurgitant fraction (RF) of 50% if regurgitant volume at the mitral valve (MV_RegVol_) is about 0.25 ml. The normal LVSV_tot_ of an elephant heart is about 20 l [62] resulting in a RF of about zero, if MV_RegVol_ is about 0.25 ml. An RF of about 50% needs an MV_RegVol_ of about 5 l

The quantitative approach is based on the determination of the individual RF. This parameter is included in all current recommendations [3, 5, 6]. RF relies on the determination of LVSV_tot_ and LVSV_eff_. The absolute value of MV_RegVol_ should always be interpreted with respect to LVEDV. It is obvious that the amount of MV_RegVol_ is much more important in small hearts than in larger hearts, which can be impressively illustrated by interspecies comparisons (Fig. 6). In consequence, haemodynamic conditions can be characterized by plausible LVEDV, LVEF, and LV forward stroke volume (= LVSV_eff_). Determination of MV_RegVol_ by the 2D-PISA method alone was associated with significant overestimation of MR_RegVol_ as documented in recent transcatheter MV repair (TMVR) trials [10, 58, 59] and further MR outcome trials [60].

**Fig. 7:**
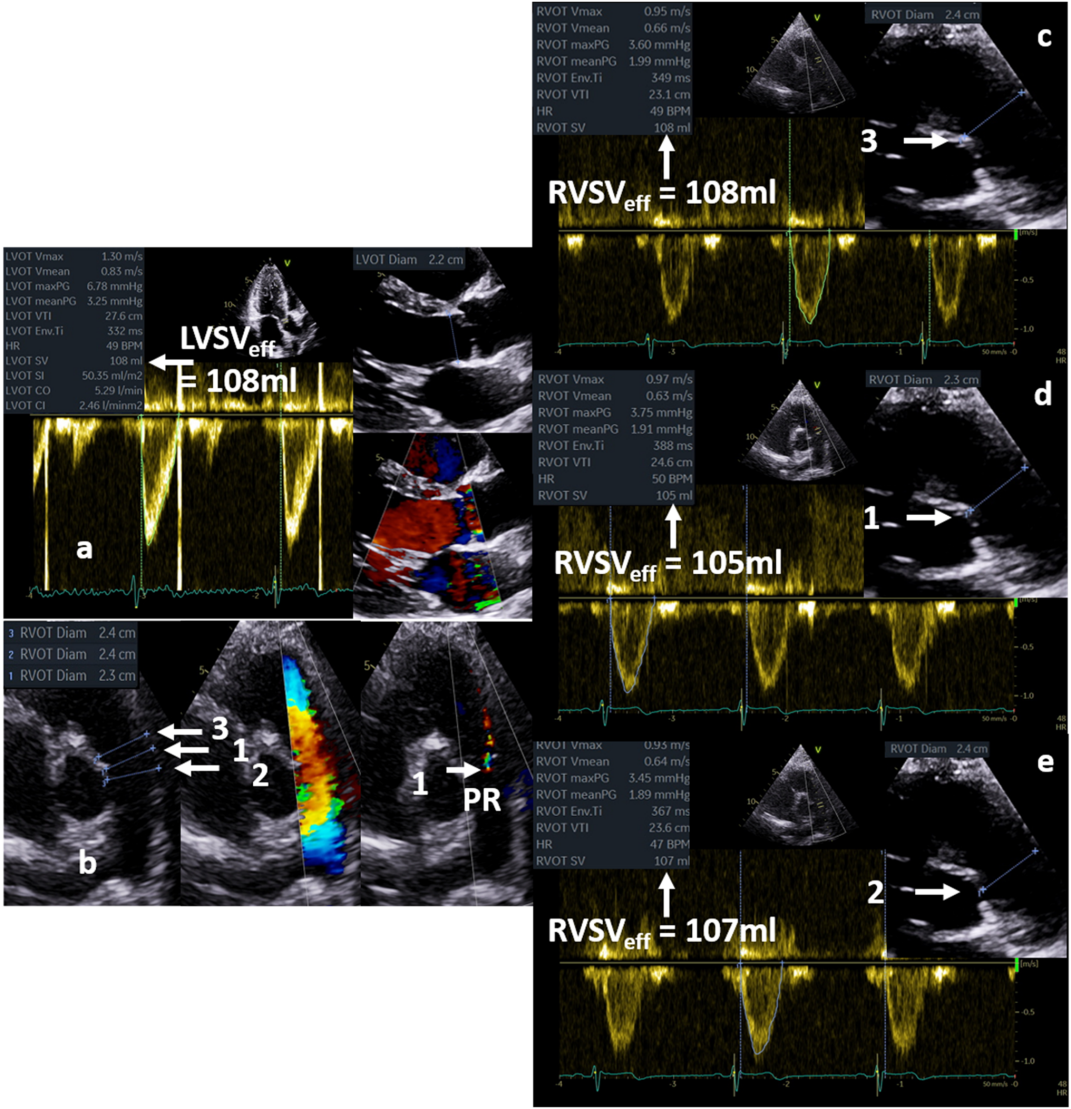
Illustration of practical aspects of LVSV_eff_ or RVSV_eff_ determination. Labeling of the D_LVOT_ and correct positioning of the pw-sample volume documented by the cusp artefact in the pw-Doppler spectrum with the respective results (**a**); three-point labeling of diameters at the level of the pulmonic valve (1) documented by the origin of the pulmonary regurgitation, at the level of the proximal pulmonic trunk (2) and at the level of the distal RVOT (3) for the respective position of the pw-Doppler sample volume (**b**); labeling of the D_RVOT_ and the corresponding pw-Doppler spectrum at the RVOT (**c**), at the pulmonic valve (**d**), and at the proximal pulmonic trunk (**e**) with the respective results. All determined forward stroke volumes are within similar ranges, hence documenting plausible results

Calculation of RF is based on the measurement of LVEDV and LV endsystolic volume (LVESV) as well as LVSV_eff_ and MV_RegVol_ to estimate cardiac output (CO) and cardiac index (CI) by echocardiography. Practical tips to avoid pitfalls when determining cardiac volumes—especially LVSV_tot_, LVSV_eff_, and right-ventricular (RV) stroke volume (RVSV_eff_), are listed in Table 6. The practical approach to check Doppler measurements of RVSV_eff_ by a plausibility cross-check is illustrated in Fig. 7. However, this concept is still not validated by prognostic data [2, 3, 5]. Compared to cardiac magnetic resonance (CMR) tomography, a significant underestimation of LV volumes by echocardiography has been reported [63]. Furthermore, over- and underestimation of LV volumes in humans [64] and phantoms [65, 66] have been described comparing different imaging methods, e.g., native 2D- and 3D echocardiography, contrast echocardiography, CMR, and computed tomography. Recently, conclusive LV volume assessment by 2D echocardiography was illustrated if image quality is adequate [67–70]. The differences in LV volumes between 2D echocardiography and CMR can be minimized by triplane, 3D-, and contrast echocardiography [71, 72]. A Doppler echocardiographic approach to calculate LVSV_tot_ by the LV filling volume has been proposed in recent recommendations using MV diameter in the 4-chamber view and the transmitral velocity time integral (VTI) at the level of the mitral annulus [2, 3, 5]. However, this approach seems to be error-prone due to the non-circular shape of the MV annulus.

**Table 6:** Target parameters of left-ventricular (LV) volumes and mitral regurgitant volume (MV_RegVol_), the different methods for assessment, the methodological limitations, and the conditions when to use or not to use the respective method

Target parameter	Methods	Limitations	When to use or not to use
LVSV_tot_	LV planimetry (2D) Monoplane long axis view (LAX) Biplane 2- and 4- chamber view (2- and 4-ChV) Triplane LV volumetry (3D) Mitral inflow (Doppler)	LV planimetry (2D) not-sufficient standardization of the views not-sufficient imaging conditions of endocardial contours foreshortening views regional wall motion abnormalities LV volumetry (3D) not-sufficient image quality, especially spatial resolution Mitral inflow (Doppler) Mitral annulus is not circular Transmitral pw-Doppler spectrum must be acquired at mitral annulus level Position of the sample volume cannot be standardized due to the movement of the mitral annulus	LV planimetry (2D)— in general, only to use if endocardial contours can be adequately delineated. If not, try to use LV opacification with contrast echocardiography. Delineation of all trabecula as endocardium causes underestimation, delineation of the midmyocardial contour between longitudinal and circumferential fibers causes overestimation of LV volumes. Carefully labeling of the apex of the cavity, the mitral annulus and the LVOT—especially wrong labeling of the basal regions produces significant underestimation of LV volumes Monoplane LV planimetry is only applicable if no wall motion abnormalities are present. Monoplane LAX planimetry results mostly in larger LV volumes in comparison to 2- and 4-ChV. Monoplane LV planimetry is misleading in patients with regional wall motion abnormalities Biplane 2- and 4-ChV is not allowed in foreshortening and not-standardized views. Thus, it is only applicable if maximum LV length is accurately documented. Monoplane 2-ChV planimetry results mostly in the lowest LV volumes, monoplane 4-ChV planimetry results mostly in the underestimated LV volumes due to foreshortening. Biplane LV planimetry is misleading in patients with regional wall motion abnormalities Triplane is the best approach to document standardized views. Triplane LV planimetry is an acceptable approach to assess reliable LV volumes in patients with regional wall motion abnormalities. Triplane LV planimetry is superior to LV volumetry (3D) in patients with not optimal image quality LV volumetry (3D)—This approach is the best one—especially in patients with regional wall motion abnormalities. However, it can only be used in patients with excellent image quality and sufficient temporal resolution (volume rates > 20/s). If volume stitching is needed, image acquisition requires regular heart rate and cooperation of the patient during breath hold Mitral inflow (Doppler)—in clinical practice this method is too error-prone to be recommended because diameter of the mitral annulus is not exactly determined in the 4-ChV and cannot be corrected with respect to the dynamic alterations during diastole. Transmitral pw-Doppler spectrum at the level of the mitral annulus must be aligned to the inflow velocities. This approach is generally obsolete in patients with mitral valve stenosis or pathologically increased transmitral velocities
LVSV_eff_	Doppler calculation using LVOT diameter (D_LVOT_) and LVOT velocity time integral (VTI_LVOT_): LVSV_eff_ = 0.785 × D_LVOT_^2^ x VTI_LVOT_	Oblique labeling of D_LVOT_ mostly causing underestimation of D_LVOT_ and LVSV_eff_ Wrong position of the position of the sample volume. If it is located too far into the left ventricle, LVSV_eff_ is underestimated	LVSV_eff_ assessment by Doppler echocardiography is well applicable in patients with normal morphology of aortic valve and LVOT LVSV_eff_ assessment by Doppler echocardiography is not applicable in patients with relevant aortic stenosis (overestimation of LVSV_eff_ due to increased VTI_LVOT_ because of flow increase proximal to the aortic valve stenosis) and/or relevant aortic valve regurgitation (overestimation of LVSV_eff_ due to increased VTI_LVOT_ which represent the addition of LVSV_eff_ and regurgitant volume at the aortic valve) If D_LVOT_ cannot be accurately measured in TTE, D_LVOT_ or cross-sectional LVOT area can be determined by 2D- or 3D-TOE imaging
RVSV_eff_	Doppler calculation using RVOT diameter (D_RVOT_) and RVOT velocity time integral (VTI_RVOT_): RVSV_eff_ = 0.785 × D_RVOT_^2^ x VTI_RVOT_	Wrong labeling of D_RVOT_ mostly caused by lung shadowing causing underestimation of D_RVOT_ and RVSV_eff_ Wrong labeling of D_RVOT_ too far into the right ventricle causing severe overestimation of D_RVOT_ and RVSV_eff_ Wrong position of the position of the sample volume in relation to the labeling of D_RVOT_. Causing both over- or underestimation of RVSV_eff_	RVSV_eff_ assessment by Doppler echocardiography is well applicable in patients with normal morphology of pulmonary valve and RVOT. Plausibility control assessment is recommended comparing measurements at different levels at the RVOT, the pulmonary valve and the pulmonary trunk (see Fig. 4) RVSV_eff_ assessment by Doppler echocardiography is not applicable in patients with relevant pulmonic stenosis or regurgitation In patients with aortic valve disease, RVSV_eff_ assessment by Doppler echocardiography (if pulmonary valve is normal and no or mild regurgitation is present) enables the estimation of LVSV_eff_ because during these conditions RVSV_eff_ is equal to LVSV_eff_ If D_RVOT_ cannot be accurately measured in TTE, D_RVOT_ or cross-sectional RVOT area can be determined by 2D- or 3D-TOE imaging
2D-PISA-MR_RegVol_	2D-PISA-method	underestimation of RegVol_MV_ by the elliptical shape of EROA overestimation by improper labeling of the PISA radius, PISA elongation by constrained flow field or eccentric jets, and by the dynamic nature of the MR; very limited, if applied in eccentric jets—even using angle correction; limited by error-proneness of the PISA radius detection	MR_RegVol_ by the 2D-PISA method is only applicable in patients with mitral regurgitation if regurgitant jet formation is not eccentric and proximal convergence areas are flat, e.g. in patients with mitral valve regurgitation type Carpentier I with reduced LV function Highly error-prone in primary MR with eccentric jet formation Not applicable in the presence of relevant mitral valve stenosis Not applicable in the presence of concomitant aortic valve diseases
Calculated MR_RegVol_	Calculation using LVSV_tot_ assessment by planimetry or volumetry and LVSV_eff_ by Doppler echocardiography: MR_RegVol_ = LVSV_tot_—LVSV_eff_	In principle, error-prone due to the assessment of multiple parameters for both, LVSV_tot_ and LVSV_eff_ determination The validity of this approach is highly dependent on image quality, standardization, technical skill, and expertise	LVSV_tot_ assessment can only be performed in native 2D echocardiography if image quality is adequate. Otherwise contrast echocardiography is recommended The choice of method for LVSV_tot_ assessment depends on alterations of LV geometry due to regional wall motion abnormalities. If image quality is adequate, 3D volumetry is superior to triplane. Triplane LV planimetry is superior to biplane. Biplane LV planimetry is superior to monoplane LVSV_eff_ assessment requires the correct position of the sample volumes of pw Doppler and the correct allocation of the respective diameters of LVOT and RVOT to the positions of the sample volume. Alternatively, diameters and cross-sectional areas can be determined by 2D- and 3D-TOE data sets

*LVSV*_*tot*_ total LV stroke volume, *LVSV*_*eff*_ effective forward LV stroke volume, *RVSV*_*eff*_ effective forward RV stroke volume, *LVOT—LV* outflow tract, *RVOT *right-ventricular outflow tract, *TOE *transoesophageal echocardiography, *TTE *transthoracic echocardiography

LVSV_eff_ in “pure” MR can be determined by Doppler calculations using cross-sectional area (CSA) or diameters of the LV outflow tract (LVOT) and the corresponding pulsed wave (pw) Doppler velocity time integral (VTI) [2, 3, 5]. In patients with combined aortic valve disease, LVSV_eff_ assessment is more complex, because Doppler calculations of LVSV_eff_ should be performed using the CSA or diameter of the RV outflow tract (RVOT) and the respective pw-Doppler VTI to assess RVSV_eff_, which corresponds to LVSV_eff_, if no or only mild pulmonary regurgitation is present. However, RVSV_eff_ measurement is challenging due to the variable anatomy of the RVOT and the additional time needed for precise measurements.

**Fig. 8:**
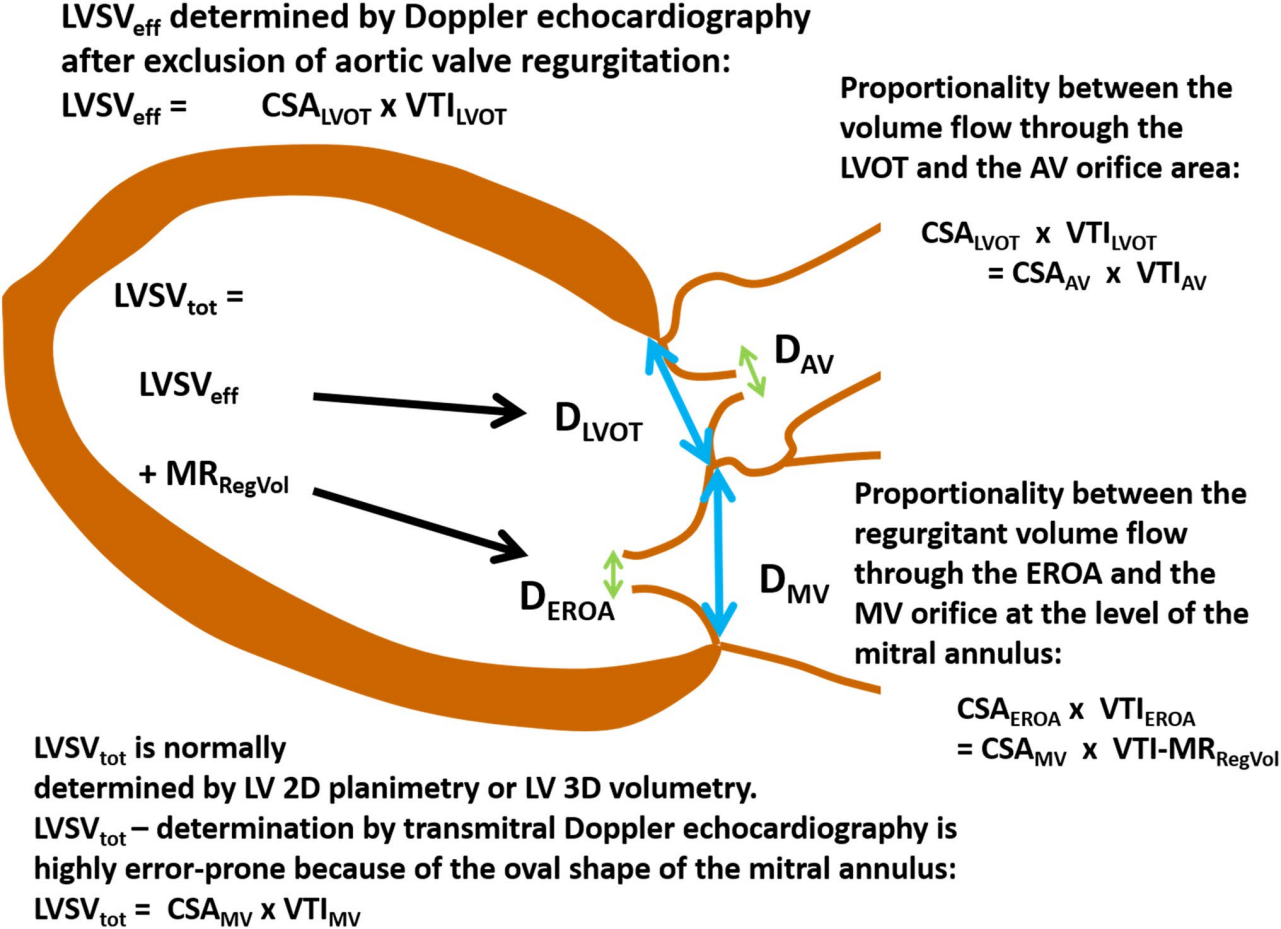
Illustration of the proportionality of forward blood flow volume or effective left-ventricular stroke volume (LVSV_eff_) and of transmitral regurgitant volume (MV_RegVol_) between the respective cross-section areas (CSAs) and blood flow velocities in a system of communicating tubes. Considering the volume flow during one heart cycle total left-ventricular stroke volume (LVSV_tot_) is the summation of LVSV_eff_ and MV_RegVol_. LVSV_eff_ at the level of the left-ventricular outflow tract (LVOT) is equal to the level of the aortic valve (AV) orifice according to the continuity equation. By analogy MV_RegVol_ at the level of the effective regurgitant orifice area (EROA) is equal to MV_RegVol_ at the level of mitral valve (MV) annulus. Thus, both LVSV_eff_ and MV_RegVol_ exhibit proportionality between respective cross-section areas (CSA) and velocity time integrals (VTI). *CSA*_*AV*_ CSA of the AV orifice, *CSA*_*EROA*_ CSA of the MV regurgitant orifice, *CSA*_*LVOT*_ CSA of the LVOT, *CSA*_*MV*_ CSA at the level of the MV annulus, *D*_*AV*_ diameter of the AV orifice, *D*_*EROA*_ diameter of the MV regurgitant orifice, *D*_*LVOT*_ diameter of the LVOT, *D*_*MV*_  diameter at the level of the MV annulus, *VTI*_*AV*_ VTI of the systolic forward blood flow through the AV orifice, *VTI*_*EROA*_ VTI of the diastolic backward blood flow through the MV regurgitant orifice, *VTI*_*LVOT*_ VTI of the systolic forward blood flow through the LVOT, *VTI*_*MV*_ VTI of the diastolic forward mitral flow at the level of the MV annulus, *VTI-MV*_*RegVol*_ VTI of the systolic regurgitant transmitral blood flow at the level of the MV annulus

**Fig. 9:**
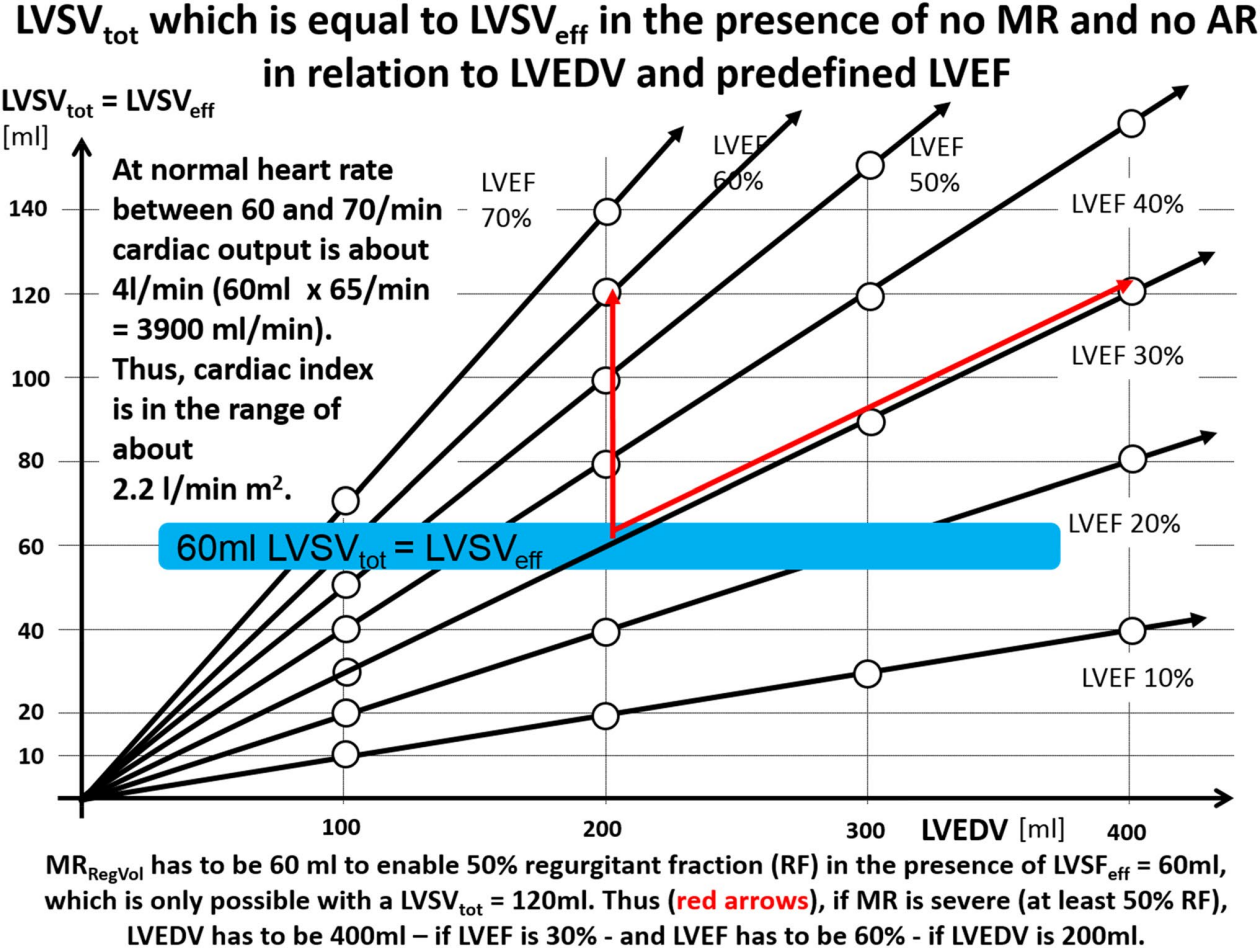
The relation between LVSV_tot_, which is equal to LVSV_eff_ in the absence of mitral regurgitation (MR) and aortic regurgitation (AR), and left-ventricular end-diastolic volume (LVEDV) with respect to left ejection fraction (LVEF) If LVEDV of 200 ml in the presence of LVEF of 30% is assumed at stable haemodynamic conditions labeled by the blue area ( LVSV_tot_ = LVSV_eff_, = 60 ml indicating a cardiac index > 2.2 l/min m^2^ at a normal heart rate of 65/min), LVSV_tot_ must be equal to LVSV_eff_, indicating the absence of MR and AR to provide the necessary cardiac output or cardiac index. The red arrows display the necessary increase of LVEDV or LVEF assuming severe MR with a regurgitant fraction of 50%. Thus, to provide LVSV_eff_ of 60 ml and MV_RegVol_ of 60 ml, LVSV_tot_ of 120 ml is necessary. Consequently, LVEDV must be 400 ml if LVEF is 30%, and LVEF must be 60% if LVEDV is 200 ml

The problem of incongruent haemodynamic measurements in MR patients is highlighted by the recently introduced terms “proportionate” and “disproportionate” MR [40, 73–75]. The concept of proportionality between blood flow and orifice areas can be illustrated by the continuity equation determining effective orifice area in patients with aortic valve stenosis (AS) [36, 76]. The same principle of proportionality can only theoretically be applied to the calculation of the MV_RegVol_ (Fig. 8), because MV_RegVol_ cannot be practically measured by pw-Doppler techniques due to methodological limitations. However, a plausibility cross-check of LVSV_tot_, LVSV_eff_, MV_RegVol_,CO, and CI can be performed independently of the method used for determination of these parameters, because proportionality is a prerequisite between EROA and MV_RegVol_. The usage of the continuity equation for MV_RegVol_ determination is impossible due to the high transmitral velocities of regurgitant flow at the level of the mitral annulus, the EROA changes of the valve during the systolic time interval, and the deceleration of flow velocities between EROA and the mitral annulus level. Because of the rheological need of proportionality between EROA and retrograde volume flow or flow velocities, the term “disproportionateness” [40, 73–75] can only be interpreted as a characterization of SMR severity in relation to the impaired LV function. However, the potential therapeutical benefit of MR treatment in relation to heart failure cannot be described by the disproportionality between LVEDV and EROA, because these parameters are proportionally interrelated at a defined LVEF (Fig. 9).

MR severity can be assessed as mild or moderate in heart failure patients at rest during compensated stage with OMT. However, this MR characterization at rest might not describe the individual risk of re-decompensation. Thus, in these cases, haemodynamic impairment should predominantly be documented by increase in SMR severity during mild-to-moderate dynamic stress testing to support this hypothesis [77, 78]. Early treatment of SMR is comprehensible during these conditions because of the potential for reverse LV remodeling and prevention of further deterioration of LV function, which should be documented by prospective trials.

## Summary and conclusion

The analysis of MR severity has become more and more important with respect to therapeutic options for MR treatment. The grading of MR severity by “eyeballing” and the 2D-PISA method is common in clinical practice, but it often leads to incongruent results with a high inter-observer variability. In addition, the dynamics of MR due to volume conditions, heart rhythm, and respective medical treatment require a high level of standardization in echocardiography. However, echocardiography allows for reliable assessment of LVSV_tot_, LVSV_eff_, MV_RegVol_, and RF in MR patients. Prerequisites are verifiable documentations, respective technical skill, and plausible measurements. The present proposal provides a “new” haemodynamically oriented workflow, which integrates a detailed MR classification scheme, considering the clinical complaints, the chronicity of the disease process, the MV morphology, and the echocardiographic parameters characterizing LA and LV remodelling. The essential point to note is the integration of a quantitative assessment of MR severity into the recent “integrated approach” to provide haemodynamic plausibility and to avoid inconsistencies of echocardiographic measurements.

## Appendix: Conference discussion

*Prof. Dr. Andreas Hagendorff (Leipzig)* The “2. Mitteldeutscher Echokardiographie Kongress” in Leipzig had the thematic priority about echocardiography in MR patients at the debate “echocardiography-guided clinical trials: TMVR trials and their consequences” and the session “quantification of MR severity: a problem—which cannot or can be solved”. Due to the corona crisis, we had to communicate mostly in T-conferences during the year 2020. Thus, this year, the discussion about the topic of our expert proposal was also web-based like the congress itself in October 2020. To introduce into the following conference discussion: Please, tell us the most frequent errors assessing MR severity using the “integrative approach”?

*Prof. Dr. Fabian Knebel (Berlin)* The most frequent error in the assessment of MR severity is the misuse of the MR jet area and its relation to the LA size. This approach is in principle inappropriate, because misinterpretation is inevitable. Further frequent errors are the use of improper images for MR assessment anyway, and the overuse of the 2D-PISA method for all MR types irrespectively to the eccentricity of the jet formation. Several simple questions can help to scrutinize the results and to uncover misinterpretations, e.g., is a relevant chronic MR possible, if the patient has no complaints?—Is a relevant MR possible in a patient with a normal LV function, if E-wave velocity is low or A-wave velocity is high?—Is a relevant MR possible if E/E` and sPAP are normal?

*Prof. Dr. Andreas Hagendorff (Leipzig)* The 2D-PISA method is highly prone to errors. However, do you think the 2D-PISA method can be accepted as a reasonable approach under certain conditions? Will 3D-PISA measurements will improve the assessment of MR severity?

*Dr. Jan Knierim (Berlin)* Notably, only the elliptical shape of the orifice area is often mentioned as a reason for underestimation of EROA and MR_RegVol_ by the 2D-PISA-method. The multiple other factors for overestimating EROA and MR_RegVol_, e.g., improper labeling of the PISA radius, PISA elongation by constrained flow field and eccentric jets, dynamic nature of the MR, etc. are rarely mentioned. However, the 2D-PISA method is quite reasonable in cases of circular EROA shape and flat PISA hemispheres like in SMR Carpentier type I patients with reduced LV function and central regurgitant jet formation. 3D-PISA is a very promising tool and will obviously solve several problems of the 2D-PISA-method. Unfortunately, it is still too time-consuming at present in daily routine.

*Prof. Dr. Andreas Hagendorff (Leipzig)* Focusing on methodological problems by echocardiography to assess MR severity, should we provide the machine settings—especially for color Doppler images—in routine practice and/or in scientific papers due to better transparency of a verifiable documentation?

*Dr. Andreas Helfen (Lünen)* Considering the methodical aspects of spectral and color Doppler data acquisition, it is desirable that even in clinical practice, ultrasound settings are provided to allow insights into the quality of Doppler measurements when therapeutic options for MR patients are discussed in the heart team. Furthermore, disease progression or improvement, e.g., due to reverse LV remodeling, can be better detected comparing images with the same settings during follow-up visits. In scientific papers, the information about the machine settings should be recommended as mandatory. A second important methodological problem is linked to the measurement of the 2D-PISA radius. Although it is stated that the radius of the proximal flow convergence has to be measured “from the point of the color aliasing to the VC” [5], all studies cited in this recommendation measured the radius from the point of color aliasing to the level of the regurgitant orifice.

*Prof. Dr. Andreas Hagendorff (Leipzig)* Do you want to propose some ideas to provide better transparency of the echocardiographic documentation of MR by color Doppler?

*Dr. Stephan Stöbe (Leipzig)* This is almost an unfair question, because it gives the impression to impose requirements on routine echocardiography. However, especially in the scientific arena, we should aim for a higher standard. Thus, it would be desirable to illustrate—e.g., if a 2D-PISA-measurement is performed—the correlation of color-coded images to the cardiac cycle by a proper ECG documentation, to present an anatomical colored M-Mode to verify the optimal alignment of central jet formation within the cardiac cycle, to perform an adjustment of color-coded Doppler settings by comparison of the color delineation at the LVOT during systole in comparison to LV wall, and to prove the sample volume position and the cursor alignment of PW and CW Doppler spectra using duplex mode. All these documentations could be transparently displayed by image libraries in scientific papers as supplementals.

*Prof. Dr. Andreas Hagendorff (Leipzig)* Comparable to the previous question, how to improve the MR analysis with respect to the time-dependency within the cardiac cycle?

*Prof. Dr. Dariusch Haghi (Ludwigshafen)* As mentioned in the paper, dynamic MR time-dependency can conventionally be documented by colour-coded anatomical M-Mode showing alterations in PISA radii during systole. Perhaps 3D-colour-coded echocardiography will improve MR analysis with respect to its time-dependency within the cardiac cycle.

*Prof. Dr. Andreas Hagendorff (Leipzig)* Beside the time-dependency of MR within the cardiac cycle, the dynamic nature of MR during various conditions—especially in SMR—should be considered in our therapeutic decision-making. Can you recommend a strategy to clarify the importance of SMR in patients, in whom the grading of MR severity at baseline cannot explain the severity of complaints?

*Dr. Nicolas Merke (Berlin)* It is important to prove that the clinical symptoms are originated from the MR. Especially in SMR, which is characterized by the diseased left ventricle, mild-to-moderate dynamic stress will increase the MR degree. Thus, dynamic stress echocardiography seems to be the adequate method to document the risk of LV failure due to SMR.

*Prof. Dr. Andreas Hagendorff (Leipzig)* The assessment of MR severity should be performed at compensated conditions during optimized treatment (revascularization, resynchronization, and OMT). How to document this prerequisite—especially in clinical trials?

*Dr. Nicolas Merke (Berlin)* The information about medication and dosages like circulatory parameters may be added to the report to ensure comparability of echocardiographic findings during OMT.

*Prof. Dr. Andreas Hagendorff (Leipzig)* The grading of MR severity during OMT arises the question about the volume state during echocardiography. What is the “physiological” volume load?

*Dr. Roland Brandt (Bad Nauheim)* The “physiological” volume load is definitively not the condition in the theatre, because normally the patient is in an almost hypovolemic state prior to invasive diagnostics or intervention. In addition, general anesthesia and positive-pressure ventilation cause a reduction in sympathetic tone and have an unloading effect on the left ventricle. Therefore, TOE under general anesthesia may underestimate the degree of MR compared to the baseline evaluation in the ambulatory setting. The so-called “physiological” volume should reflect real-life conditions. Thus, we propose the echocardiographic analysis 1 day prior to intervention or surgery and at hospital discharge at comparable cardiovascular conditions for objective documentation of treatment effects.

*Prof. Dr. Andreas Hagendorff (Leipzig)* With respect to possible dehydration, is it allowed to assess MR severity by inducing volume overload?

*Dr. Nicolas Merke (Berlin)* It is certainly allowed to test intraoperatively MR severity by increasing volume load. On the other hand, the volume amount to adjust a “normal” state in the catheter laboratory is not known. Thus, provoking increased MR severity by volume load or drug-induced increase of LV afterload is crucial. The better way to test the dynamics of MR severity—as mentioned before—is a dynamic stress test. Even if the semi-quantitative and quantitative parameters are difficult to assess during dynamic stress echocardiography, the pathological increase of E/E´ and sPAP can often be determined during stress and will be helpful for decision-making.

*Prof. Dr. Andreas Hagendorff (Leipzig)* The characterization of MR pathology is based on the Carpentier classification and the differentiation between PMR and SMR. Are there further aspects to classify MR with respect to potential MR treatment?

*Dr. Daniel Lavall (Leipzig)* The Carpentier classification and the differentiation between PMR and SMR are crucial for medical, surgical, and interventional therapy as well as for prognostic considerations. However, multiple aspects—the MR etiology, the MR chronicity, concomitant diseases, clinical complaints, and special echocardiographic findings—may also be considered in a classification to specify more in detail the underlying MR mechanisms. We assume that the proposed classification aligned to echocardiographic and clinical characteristics might be helpful to guide therapeutic approaches.

*Prof. Dr. Andreas Hagendorff (Leipzig)* How to define the morphological differences between fibroelastic deficiency, Barlow`s disease and MV annulus disjunction?

*Dr. Tobias Ruf (Mainz)* Fibroelastic deficiency is characterized by thin MV leaflet, MV prolapse—frequently in a single scallop—and frequent chordae rupture, whereas Barlow`s disease shows bulky billowing leaflets with excess of tissue, thick MV leaflets and multi-segmental prolapse. However, the echocardiographic characterization is challenging. In contrast the MV annulus disjunction is a sole echocardiographic diagnosis showing insertion of the MV leaflets at the LA level and a curling motion of the LV wall during systole predominantly in the posterolateral LV segments.

*Prof. Dr. Andreas Hagendorff (Leipzig)* Again, focusing on MV morphology do you think that 3D echocardiography is mandatory for better diagnosis of leaflet perforation or clefts?

*PD Dr. Ertunc Altiok (Aachen)* The answer is easy—“yes”. 3D TTE and 3D TOE allow improved en-face visualizing of MV leaflets permitting more precise localization of pathologies and the origin of regurgitation jets compared to 2D echocardiography. However, it should be noted that 3D TOE imaging can be limited particularly by dropout artefacts giving the appearance of not existing valve perforation or cleft. This limitation may be overcome by visualization of the defect in more than one view and by demonstration of color Doppler flow through the valve at the site of the suspected perforation or cleft.

*Prof. Dr. Andreas Hagendorff (Leipzig)* Looking at the individual RF as an objective individual parameter of MR severity—is this quantitative approach a solution to provide conclusive echocardiographic results?

*PD Dr. Sebastian Ewen* The accurate quantification of MR severity by the individual RF would be the desirable “gold standard” of MR assessment. However, this approach is the most difficult one and requires maximum accurateness of echocardiographic image acquisition and high expertise of measurement procedures. Thus, the quantitative RF determination is comparably error-prone as the “integrated approach” of the current recommendations [3, 5] in untrained echocardiography. The verification of cardiac volumes determined by Doppler techniques or planimetry/volumetry—especially the comparison of LVSV_tot_, LVSV_eff_, and MR_RegVol_ in MR patients—is necessary to provide haemodynamic plausibility and to avoid miscalculation of MR severity.

*Prof. Dr. Andreas Hagendorff (Leipzig)* The quantitative approach of MR assessment is still a maximum challenge. Do you think this approach can be implemented into the so-called clinical routine?

*Prof. Dr. Fabian Knebel (Berlin)* My answer will disappoint you. The determination of the individual RF is nothing for routine scanning procedures. In addition, we need a complete rethinking in echocardiography regarding the possibilities and options of accurate LV and RV volume assessment by all possible echocardiographic techniques to accept this quantitative approach. However, if AS patients are classified according to flow conditions determined by Doppler echocardiography. Perhaps, it is possible to determine LVSV_eff_ in MR patients, and thus, the estimation of CO and CI might be the first step to characterize the cardiovascular conditions in MR patients. Currently, the quantitative approach of RF determination is only feasible for experts.

However, to finalize our discussion, what is the most important challenge to improve the quality of echocardiography for assessment of MR severity?

*Prof. Dr. Andreas Hagendorff (Leipzig)* The answer is simple. We need to improve echocardiographic teaching as concerns basic knowledge of physics, anatomy and pathophysiology, technical skill of the scanning, image optimization, standardization and completeness of documentation, clinical experience, interpretation of the findings, and knowledge about possible treatment options. I think, we have still to cover these educational challenges—even in the near era of artificial intelligence.
